# Comparison of Tensile Bond Strength of Fixed-Fixed Versus Cantilever Single- and Double-Abutted Resin-Bonded Bridges Dental Prosthesis

**DOI:** 10.3390/ma15165744

**Published:** 2022-08-19

**Authors:** Shweta Narwani, Naveen S. Yadav, Puja Hazari, Vrinda Saxena, Abdulrahman H. Alzahrani, Ahmed Alamoudi, Bassam Zidane, Nasreen Hassan Mohammed Albar, Ali Robaian, Sushil Kishnani, Kirti Somkuwar, Shilpa Bhandi, Kumar Chandan Srivastava, Deepti Shrivastava, Shankargouda Patil

**Affiliations:** 1 Department of Prosthodontics and Crown & Bridge and Implantology, Peoples Dental Academy, Peoples University, Bhopal 462037, India; 2 Department of Public Health Dentistry, Government Dental College, Indore 452001, India; 3 Department of Prosthodontics, Faculty of Dentistry, Taif University, Taif 21944, Saudi Arabia; 4 Department of Oral Biology, King Abdulaziz University, Jeddah 80200, Saudi Arabia; 5 Department of Restorative Dentistry, King Abdulaziz University, Jeddah 22254, Saudi Arabia; 6 Department of Restorative Dentistry, Jazan University, Jazan 45142, Saudi Arabia; 7 Department of Conservative Dental Sciences, College of Dentistry, Prince Sattam bin Abdulaziz University, Al-Kharj 16278, Saudi Arabia; 8 Department of Conservative Dentistry & Endodontics, Peoples College of Dental Sciences, Peoples University, Bhopal 462037, India; 9 Department of Oral & Maxillofacial Surgery & Diagnostic Sciences, College of Dentistry, Jouf University, Sakaka 72388, Saudi Arabia; 10 Department of Preventive Dentistry, College of Dentistry, Jouf University, Sakaka 72388, Saudi Arabia; 11 Department of Maxillofacial Surgery and Diagnostic Sciences, Division of Oral Pathology, College of Dentistry, Jazan University, Jazan 45142, Saudi Arabia

**Keywords:** properties, bond strength, debonding, dental prosthesis, resin-bonded, fixed prosthesis, cantilever, fixed-fixed, Maryland bridge, Rochette bridge

## Abstract

Resin-bonded fixed dental prostheses (RBFDP) are minimally invasive alternatives to traditional full-coverage fixed partial dentures as they rely on resin cements for retention. This study compared and evaluated the tensile bond strength of three different resin-bonded bridge designs, namely, three-unit fixed-fixed, two-unit cantilever single abutment, and three-unit cantilever double-abutted resin-bonded bridge. Furthermore, the study attempted to compare the tensile bond strengths of the Maryland and Rochette types of resin-bonded bridges. Based on the inclusion and exclusion criteria, a total of seventy-five extracted maxillary incisors were collected and later were mounted on the acrylic blocks. Three distinct resin-bonded metal frameworks were designed: three-unit fixed-fixed (n = 30), two-unit cantilever single abutment (n = 30), and a three-unit cantilever double abutment (n = 30). The main groups were further divided into two subgroups based on the retainer design such as Rochette and Maryland. The different prosthesis designs were cemented to the prepared teeth. Later, abutment preparations were made on all specimens keeping the preparation as minimally invasive and esthetic oriented. Impression of the preparations were made using polyvinyl siloxane impression material, followed by pouring cast using die stone. A U-shaped handle of 1.5 mm diameter sprue wax with a 3 mm hole in between was attached to the occlusal surface of each pattern. The wax patterns were sprued and cast in a cobalt–chromium alloy. The castings were cleaned by sandblasting, followed by finishing and polishing. Lastly, based on the study group, specimens for Rochette bridge were perforated to provide mechanical retention between resin cement and metal, whereas the remaining 15 specimens were sandblasted on the palatal side to provide mechanical retention (Maryland bridge). In order to evaluate the tensile bond strength, the specimens were subjected to tensile forces on a universal testing machine with a uniform crosshead speed. The fixed-fixed partial prosthesis proved superior to both cantilever designs, whereas the single abutment cantilever design showed the lowest tensile bond strength. Maryland bridges uniformly showed higher bond strengths across all framework designs. Within the limitations of this study, the three-unit fixed-fixed design and Maryland bridges had greater bond strengths, implying that they may demonstrate lower clinical failure than cantilever designs and Rochette bridges.

## 1. Introduction

Loss of teeth is generally an unpleasant outcome for patients, and it can be considered as a reflection of the patient’s history of dental conditions and treatments. According to the estimates from the Global Burden of Disease, more than half of the world’s population experience single or partial tooth loss [1]. A person’s quality of life in terms of oral health might have a negative impact due to tooth loss [2]. The field of prosthetic rehabilitation has changed drastically as a result of advancements in high-strength ceramics and digital dentistry. Conventional procedures for the replacement of missing teeth comprises full veneer fixed partial dentures for which the preparation of abutment teeth often involves major removal of the tooth structure. Significant advancements in material sciences have made it possible to use adhesive techniques that need less invasive tooth preparation. Resin-bonded bridges (also known as resin-bonded permanent dental prostheses or resin-retained bridges) are a less intrusive treatment option than single implants. As a result, it is implied that fewer dentinal tubules are opened, which is advantageous for the long-term health and vitality of teeth [3]. Additionally, patients may choose resin-bonded fixed dental prostheses because of cost considerations and a desire to avoid extensive tooth preparation or surgery for dental implants [4]. The endurance of resin-bonded bridges has been demonstrated by long-term clinical data [5]. They are effective and considered as a reasonably priced therapeutic option for replacing teeth.

Resin-bonded bridges (RBBs) are a type of fixed dental prostheses used to replace missing teeth. They are held in place by adhesive composite resin and are supported by abutments [6]. The Rochette bridge was the RBB’s original design, and it relied on the macromechanical nature of retention through metal retaining wings [7]. The resin rivet holding the prosthesis to the acid-etched enamel would be created by the luting cement that flows through the tapered pinholes. However, Rochette-type RBBs had a short lifespan. In order to enable micromechanical retention, Maryland bridges were created [8]. They had a metal surface that had been electrochemically etched, which allowed for improved resin bonding and higher survival rates [9].

The lifespan of resin-bonded fixed dental prosthesis (RBFDP) has been the focus of discussion since its conception [10]. According to the research on RBFDP retention, numerous factors can affect its clinical performance, including choosing the right patient, choosing the right occlusal contacts, and designing the framework. The quantity of abutments that are connected to the pontic is another element that affects the clinical retention [11].

Fixed partial dentures’ durability may be influenced by the prosthesis design [12]. For resin-bonded fixed partial dentures (RBFDP), two-unit cantilevered or three-unit fixed-fixed designs are the two common options. One of the most recommended and effective tooth replacement procedures in the past was the three-unit fixed-fixed design [13].

The usefulness of fixed-fixed bridges is constrained by complex clinical settings. As a result, there are many designs that take into account the patient’s needs, anatomical constraints, and biomechanics [14]. A cantilevered single-abutment fixed partial prosthesis can be used to treat patients with distal extension edentulous space [15]. They have several benefits such as better oral hygiene, low maintenance, and a lower propensity for dental cavities. The preservation of the tooth anatomy, ease of preparation, and fabrication are just a few additional benefits [16].

In RBFDPs, minor and varied tooth motions are inevitable [17]. A troublesome issue with cantilevered structures is unilateral debonding [18], which might be a consequence of flexible cross-section and strong peeling forces under stress. In the past, it was considered that FPDs should have two firm abutment ends [19], and thus the abutment teeth were subjected to greater demands for tooth preparation. Recent studies have refuted the conventional dogma by demonstrating that two-unit fixed cantilevered RBFDPs have comparable clinical and patient-reported outcomes to double-abutted designs [15,20]. This might be a result of complicated inter-abutment strains from the older designs.

The resin-bonded bridge’s retainer edges are placed supragingivally, which makes these prostheses more pleasing to the periodontium, eases the impression-making and finishing procedure, and makes them easier for the patient to clean. They also offer a shorter chair time, which makes them appealing to both the patient and the dentist [21]. Patients experience less dental anxiety and worry because the preparations are mainly limited to enamel and can be performed without local anesthesia [10].

Several clinical studies and systematic reviews have reported on the survivability of RBFDPs [22,23]. RBBs have a survival rate of 87.7% at five years and 64.9% at ten years [23,24]. The most frequent complication noted was debonding such as loss of retention [23,24].

On the other hand, the heterogeneous designs of the resin-bonded dental prosthesis are not scrutinized and are evaluated as a monolith. This makes it impossible to evaluate the performance of a specific design. The connection between the framework and the resin is still the most susceptible part of a resin-retained prosthesis. As a result, the primary goal of this study was to determine whether RBFDP design, Maryland or Rochette, and which type of abutment support will give the highest retention.

In summary, most of the earlier studies performed in relation to this topic are clinical trials, the results of which may be influenced by various patients’ dependent variables due to which the results may not be applicable to a wider population. So, in order to increase the authenticity, the current study was performed with an in vitro design where the variables can be standardized to find out the effect of the number of abutments and the prosthesis design on the strength of the prostheses specifically.

This study aimed to compare and evaluate the tensile bond strength of three different resin-bonded bridge designs, namely, a three-unit fixed-fixed, two-unit cantilever single abutment, and a three-unit cantilever double-abutted resin-bonded bridge. The study further compared the tensile bond strengths of the Maryland and Rochette types of resin-bonded bridges. The null hypothesis considered was that the tensile bond strength of the cantilever designs is comparable to fixed-fixed designs, and the tensile bond strength of the prosthesis will not be affected by number of abutments and design of the prosthesis.

## 2. Materials and Methods

This in vitro experimental study was conducted in the Department of Prosthodontics, Crown, and Bridge and Implantology at People’s Dental Academy, Bhopal, India, with supporting technical assistance from the Central Institute of Plastics Engineering and Technology, Bhopal, India. Prior to the study, ethical approval from the Institutional Ethics Review Board (2014/IEC/300/19) was obtained.

The specimens were divided into three groups based on support provided at the end of the prostheses. These were further allocated into two subcategories based on the design of the retainer. The resin-bonded frameworks with different prosthesis designs were cemented to the prepared teeth, and the cemented frameworks were subjected to tensile forces on a universal testing machine with a uniform crosshead speed.

### 2.1. Specimen Preparation

#### 2.1.1. Mounting of Extracted Teeth on the Acrylic Block

The inclusion criteria considered was recently extracted healthy teeth, whereas carious, unrestorable, fractured, and hypoplastic teeth were excluded from the study. A total of seventy-five sound maxillary incisors were collected after extraction from the patients with rapidly progressive periodontitis. A prior informed consent was acquired from all subjects.

Initially, the extracted teeth were stored in 10% formalin. Later, they were mounted on acrylic molds such that the roots were embedded in acrylic, whereas the crown portion above the cementoenamel junction was left exposed. The incisors were mounted in the following three different forms based on the support provided at the end of the prostheses:

(a) Fixed-fixed bridge—Two incisors were mounted such that space for a pontic was left between the two teeth. A total of 30 teeth were mounted to make 15 fixed-fixed bridge specimens.
(b) Cantilever single-abutted bridge—A single incisor was mounted in acrylic which could be used as the abutment. A total of 15 incisors were mounted in this manner to create 15 similar cantilever single-abutment bridge specimens.
(c) Cantilever double-abutted bridge—Two central incisors were placed at adjacent positions to be used as abutments. In total, 30 teeth were mounted to build 15 cantilever double-abutted bridge specimens.

**Figure 1:**
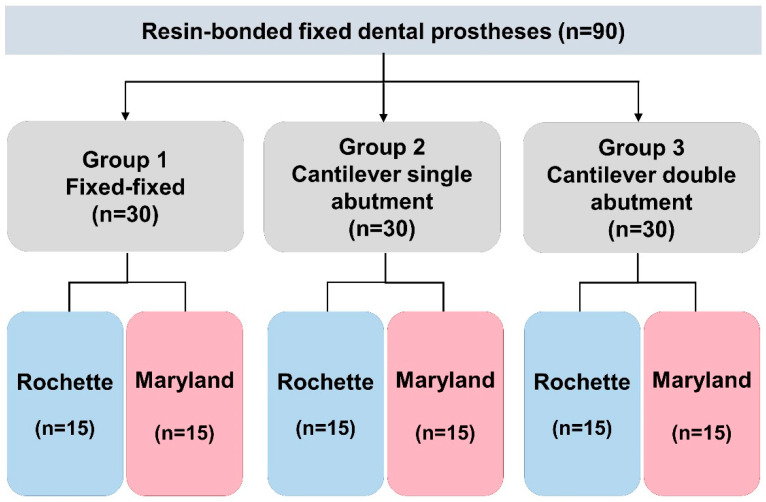
Sample Distribution.

These main groups were further divided into two subgroups based on the design of the retainer (Figure 1), such as the Rochette and Maryland types. In summary, a total of 90 prostheses were made, where 30 prostheses were made for each main group, of which 15 were kept for the Rochette type and 15 were kept for the Maryland type.

#### 2.1.2. Preparation of Incisors for the Prosthesis

The abutment teeth were prepared to adapt the retainers within the initial tooth outline keeping in mind the concern of satisfactory appearance. On the proximal aspect, the tooth preparation included axial trimming and guide planes, slightly extending onto the facial aspect to attain a facio-lingual lock. The preparation encompassed at least 180° of the teeth to augment the resistance of the retainer. The finish line of the incisor was kept 2 mm short of the incisal edge so as to prevent the incisal edge translucency from being esthetically impaired. Additionally, the lingual aspect was trimmed to produce a lingual clearance of 0.5 mm. The gingival finish line was kept 1 mm supragingival to maintain the preparation in enamel in order to ensure optimal bonding. Interproximally, the preparation was extended to the center of the contact area which maximized wraparound and at the same time minimized the visibility of metal from the facial aspect. The proximal surfaces were kept as parallel as possible to increase the retention form. Proximal grooves were added to compensate for any lack of proximal wraparound and to increase the retention form. 

#### 2.1.3. Laboratory Procedures

**Figure 2:**
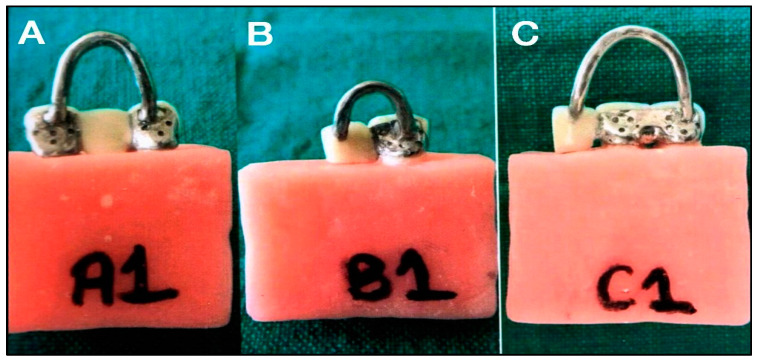
Specimens of Rochette-type resin-bonded fixed dental prostheses for (**A**) fixed-fixed, (**B**) cantilever single abutment, and (**C**) cantilever double abutted.

**Figure 3:**
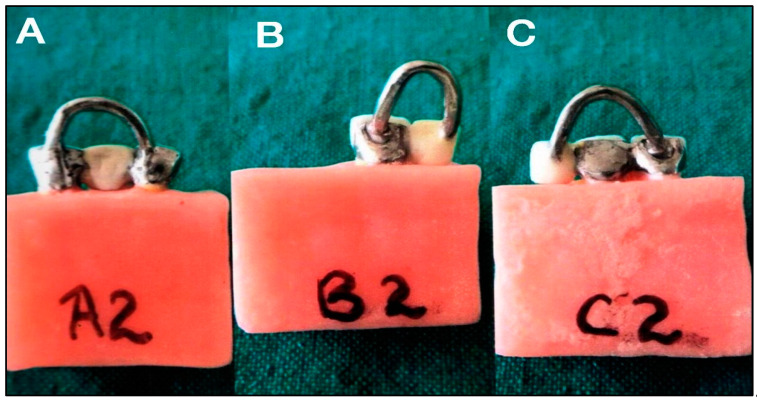
Specimens of Maryland-type resin-bonded fixed dental prostheses for (**A**) fixed-fixed, (**B**) cantilever single abutment, and (**C**) cantilever double abutted.

After tooth preparation, impressions of the preparation were made with polyvinyl siloxane impression material (3M ESPE, MN, USA). Stone dies (Die stone class IV, Kalrock, Kalabhai Dental, India) were constructed from each impression followed by wax pattern fabrication. A U-shaped handle of 1.5 mm diameter sprue wax with a 3 mm hole in between was attached to the occlusal surface of each pattern to facilitate seating and removal during subsequent stages. It also assisted in pulling the specimen with the help of fixtures during tensile bond strength testing in a universal testing machine. The wax patterns were sprued and cast in a cobalt–chromium alloy (Wironium plus, Bego). The castings were cleaned by sandblasting, followed by finishing and polishing. Fifteen frameworks from each group were perforated to provide mechanical retention between resin cement and metal (Rochette bridge) (Figure 2). The remaining 15 specimens were sandblasted on the palatal side using 50–250 µm aluminum oxide to provide mechanical retention (Maryland bridge) (Figure 3).

#### 2.1.4. Cementation and Testing of the Specimens

The frameworks were cemented using resin cement (Rely X U200 resin cement, 3M, India). Each cemented specimen was placed in a lower holder such that the tooth die was oriented with its longitudinal axis parallel to the detaching force and a hook was placed in the upper holder of the universal testing machine. This hook was entangled in the U-shaped holder attached to the casted specimen. They were subjected to tensile force on the universal testing machine (Instron-3382, Instron, MA, USA) with a crosshead speed of 1 mm/min. The maximum load required to remove the crown was measured on the universal testing machine and compared with specimens of the other study group. Each mounted specimen was cemented with a Rochette bridge, which was subjected to tensile force. This was followed by cementation of the Maryland bridge on the same specimen, and the specimen was subjected to debonding force. This procedure for tensile bond assessment was carried out on all fifteen samples of each main group, with each sample being subjected twice to the debonding force: once for the Rochette type and once for the Maryland type.

### 2.2. Statistical Analysis

The data were statistically analyzed using the Statistical Package of Social Science (SPSS software v.20; IBM Corp., Armonk, NY, USA). Kruskal–Wallis and Mann–Whitney ‘U’ tests were applied for comparing data between the study groups. The significance level was set at *p* < 0.05.

## 3. Results

**Figure 4:**
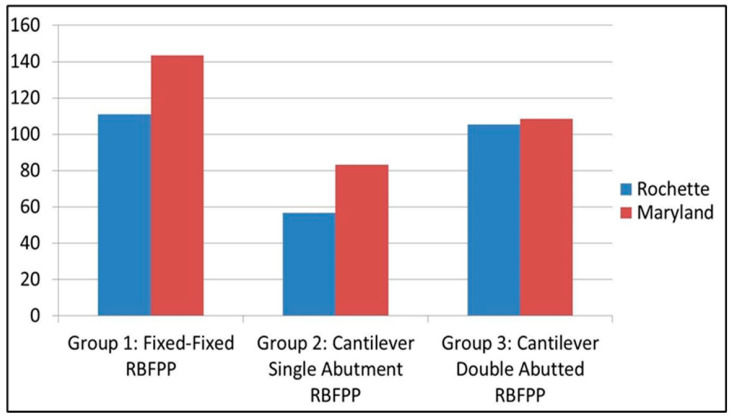
Comparison of Tensile Bond Strength (N) between Rochette and Maryland Type of Fixed-Fixed Denture, Cantilever Single and Double Abutted Resin Bonded Fixed Partial Prosthesis.

According to the results obtained, the fixed-fixed design resin-bonded fixed dental prosthesis (RBFDP) showed the greatest tensile bond strength, followed by the cantilever double abutment design (Figure 4). The cantilever single abutment design had the least tensile bond strength (Table 1). The mean tensile bond strength of fixed-fixed partial prosthesis (Group 1) was 127.23 N with a standard deviation (SD) of 21.91 N, whereas the mean tensile bond strength of cantilever single- (Group 2) and double-abutted FPD (Group 3) was 69.99 N and 106.90 N, respectively, with an SD of 30.06 N and 29.92 N (Table 1).

The Maryland design of resin-bonded fixed dental prosthesis (RBFDP) had greater tensile bond strength than the older Rochette bridge (Table 2) in two groups, i.e., fixed-fixed and cantilever single abutment RBFDP. In Group 3, i.e., cantilever double-abutted RBFDP, the Maryland type had greater tensile bond strength than the Rochette type, but the difference was non-significant. The Maryland type had a tensile strength of 143.32 ± 19.60 N compared to 111.13 ± 7.44 N in the Rochette type in fixed-fixed designs of RBFDP.

In the cantilever single-abutted RBFDP group, the Maryland type had a bond strength of 83.38 ± 35.41 compared to 56.59 ± 15.30 for the Rochette type (Table 2).

## 4. Discussion

For decades, resin-bonded bridges have been used to rehabilitate the edentulous spaces. They have a number of advantages over the traditional full-coverage fixed partial dentures, including lower costs and higher patient satisfaction [25,26], although, due to debonding, resin-bonded fixed dental prostheses (RBFDP) are more likely to fail than traditional fixed prostheses. However, the failures are often less catastrophic [27], and they do not include apical disease, migration, or cavities, which can lead to abutment loss [28,29]. The current study aimed to evaluate and compare the tensile bond strength of three desired designs of resin-bonded fixed dental prostheses based on the framework design and the number of abutments.

We found that fixed-fixed RBFDPs had the highest tensile bond strength compared to cantilever single-abutment and cantilever double-abutment RBFDPs. This suggests that fixed-fixed RBFDPs have a higher survival rate and better retention than cantilever designs. These findings are contrary to data obtained by Chai et al., who reported that two-unit RBFDPs had a better prognosis at 48–60 months with a survival rate of 81% compared to three-unit RBFDPs with a survival rate of 63% [16]. Our results are also inconsistent with findings of Wong and Botelho et al., who reported that bond strengths of the fixed-fixed group were lowered by fatigue loading. They concluded that three-unit fixed-fixed RBFDPs have a lower bond strength than two-unit cantilevered prostheses [11]. The discrepancies in outcomes may be due to the study design. Wong and Botelho et al. investigated the bond strength of framework designs bonded to stainless steel tooth dies, whereas, in the current study, the bond strength of frameworks bonded to extracted teeth were evaluated. Debonding may be connected to inter-abutment stress, functional loads, and framework biomechanics. Debonding and failure may also be caused by the framework’s substance. Metal–ceramic RBFDPs generally fail due to debonding, whereas all-ceramic RBFDPs fracture [28]. According to a thorough review by Wei et al., RBFDP longevity is dependent on the resin bond and the operator technique [30].

Our findings differ from those of Botelho et al. [31], who found that two-unit cantilevered RBFDP (CL2) designs performed much better than three-unit fixed-fixed designs (FF3). Only 50% of FF3 designs survived, but 100% of CL2 designs performed well. The greater failure rate of FF3 designs is related to differential abutment tooth motions, which cause stress at the bonding interface and debonding. The CL2 designs are not concerned with such inter-abutment stress [18]. Cantilever designs have lower biological costs and are simpler to produce, resulting in fewer difficulties than fixed-fixed structures [30].

The higher bond strength of fixed-fixed RBFDPs and cantilever double-abutted RBFDPs can be attributed to their design. Both frameworks have increased surface area due to the involvement of two abutments compared to a cantilever single abutment design. Fixed-fixed RBFDPs show greater bond strength due to their bilateral support. In cantilever double-abutment RBFDPs there is unilateral support, which may lead to stress concentration on one side only. This may lead to a greater risk of debonding with this design.

We found that cantilevered single-abutment frameworks had the lowest tensile strength. Our finding differs from a majority of previous literature that describes single-abutment cantilever designs as having a low risk of failure and greater longevity [32,33,34]. Alraheam et al. also reported that although the dental implants seem to have provided reliable support for dental restorations in recent decades, the RBFDPs can serve as a promising alternative because the estimated 5-year clinical performance of RBFDPs was similar to that of FDPs and implant-supported crowns; thus, they concluded that clinicians should consider using RBFDPs frequently [35]. In their review, Mine et al. stated that cantilever RBFDPs were found to have better prognosis as compared to two retainer RBFDPs. The explanation given by them for the inferior clinical outcome of two retainer RBFDPs was because of their two major disadvantages. Firstly, the difference in the mobility of the two abutment teeth often leads to torquing and shear forces on abutment teeth resulting in debonding of the retainer from the tooth having lesser mobility. Secondly, the differential mobility can result in unrecognized debonding, increasing their predisposition to caries [36]. The contrary results of our study could be due to the design differences. A majority of information is based on longitudinal studies, which have been poorly controlled [23]. In the in vivo study design, it may be difficult to control a single variable and several factors can influence the outcomes such as the oral environment during cementation, operator technique, and prosthesis site. The use of different cements and preparation techniques leads to heterogeneity in study designs, making it difficult to isolate factors affecting the outcome [6].

In all three configurations, we discovered that the Maryland bridge design had stronger bond strength than the Rochette bridge design. We chose air particle abrasion over standard electrochemical etching because it improves retention of metal-framework RBFDPs [37]. Our findings substantially support the findings of Berekally and Smales, who stated that the failure rate of Rochette bridges (75%) was significantly higher than that of Maryland bridges (42%). Debonding was the primary cause of their failure [38]. Similarly, Creugers et al. showed that at 7.5 years, Rochette bridges had a poor survival rate of only 28% [39].

The results were comparable to those reported by Brady et al. in 1985, when the etched discs could sustain more than four times the breaking load of the perforated discs [40]. Creugers et al. demonstrated that micromechanical retainers retained more than macromechanical retainers [41]. According to Creugers et al., the survival rates for etched metal RBBs and perforated RBBs were 78 and 63 percent, respectively [42].

The Rochette design was the first resin-bonded permanent dental prosthesis design framework. They had a significant failure rate, necessitating the Maryland bridges’ developed design. Surface treatment can alter the lifespan of resin-bonded bridges, according to el-Mowafy et al. [43]. Priest observed that chemical or electrolytic etching produces favorable long-term results, suggesting the superiority of Maryland bridges [44].

Several variables could explain why Rochette bridges performed poorly in our investigation. The retention area in RBFDPs based on macromechanical retention is limited to the perforated apertures and is not distributed evenly across the metal surface [41]. In RBFDPs based on macromechanical retention, the luting agent is introduced to the oral cavity. This allows for abrasion, fluid leakage between the metal and resin interfaces, and resin fluid absorption [41].

Our study is limited by its in vitro design. It may have failed to replicate the nuances of the oral cavity which may affect bond strength. The extracted teeth used may have had morphological differences that may contribute to variability.

Overall, our study found that fixed-fixed designs were superior to single and double abutment cantilever designs. Maryland bridges had higher bond strengths than Rochette bridges. Further research is necessary to confirm and validate these findings. Future studies may better demonstrate the clinical efficacy of resin-bonded fixed dental prostheses by including multiple operators across several prosthesis sites.

## 5. Conclusions

Replacement of missing teeth with a resin-bonded fixed dental prosthesis (RBFDP) is a conservative alternative to conventional fixed partial dentures. Various research performed in relation to RBFDP has shown that the long-term prognosis of RBFDP depends upon physical, chemical, and biological factors [45]. Within the limitations of this in vitro study, the fixed-fixed framework showed the greatest tensile bond strength. The cantilever single abutment RBFDP had the least bond strength. The Maryland bridge design of prosthesis was superior to the Rochette bridge in all three designs. Careful case selection and meticulous treatment planning are central to achieving the long-term survival of the prosthesis. Further research is needed to understand the effects of various prognostic factors.

## Figures and Tables

**Table 1:** Comparative evaluation of tensile bond strength (N) between the different resin-bonded fixed dental prosthesis.

Study Groups	Sample Size	Tensile Bond Strength			*p* Value
		Mean	SD	Median	
Group 1: Fixed-fixed denture	30	127.23	21.91	117.76	0.001
Group 2: Cantilever single abutment	30	69.99	30.06	64.26	
Group 3: Cantilever double abutment	30	106.90	29.92	114.13	

Note: Result expressed in mean; *p* < 0.01—Highly significant.

**Table 2:** Tensile bond strength (in N) of Rochette and Maryland types of resin-bonded fixed dental prosthesis (RBFDP).

		Group 1 Fixed-Fixed		Group 2 Cantilever Single Abutment		Group 3 Cantilever Double-Abutted	
		Rochette (N = 15)	Maryland (N = 15)	Rochette (N = 15)	Maryland (N = 15)	Rochette (N = 15)	Maryland (N = 15)
Tensile Bond Strength	Mean	111.13	143.32	56.59	83.38	105.27	108.52
	SD	7.44	19.60	15.30	35.41	33.48	26.98
	Median	109.56	145.56	58.27	86.59	112.21	119.30
Mann–Whitney ‘U’ Test Value		14.00		62.00		111.00	
*p* value		0.001 (HS)		0.036 (S)		0.950 (NS)	

Note: Result expressed in mean; *p* > 0.05—Not Significant; *p* < 0.05—Significant.

## Data Availability

The datasets will be available and accessible from the corresponding author on reasonable request.
